# Progress for RAD18’s mechanism in breast cancer radioresistant

**DOI:** 10.1515/biol-2025-1356

**Published:** 2026-09-08

**Authors:** Yiming Zhao, Yuanhua Liu, Yiyang Yu, Lili Cao, Yanbin Xu

**Affiliations:** School of Clinical Medicine, Shandong Second Medical University, Weifang 261053, China; Department of Oncology, Zibo Central Hospital, Zibo 255020, China; Department of Breast and Thyroid Surgery, Zibo Key Laboratory of Breast Cancer Individualized Diagnosis, Treatment and Transformation, Zibo Central Hospital, Zibo 255020, China

**Keywords:** breast cancer, RAD18, PI3K/AKT/mTOR, radiotherapy, radioresistant

## Abstract

Radioresistance remains a critical barrier to achieving durable locoregional control and improved survival outcomes in breast cancer. RAD18, an E3 ubiquitin ligase that regulates translesion synthesis and post-replication repair, has emerged as a candidate mediator of this phenotype by promoting proliferating cell nuclear antigen (PCNA) ubiquitination, facilitating replication stress tolerance, and enabling tumor cell survival following DNA damage. In parallel, the PI3K/AKT/mTOR (PAM) signaling pathway represents one of the most frequently dysregulated oncogenic networks in breast cancer and is increasingly recognized as a central determinant of therapeutic resistance. Notably, however, its biological and clinical relevance is highly context-dependent, with the strongest evidence observed in hormone receptor–positive (HR+)/HER2-negative disease and a more heterogeneous, less well-defined role in triple-negative breast cancer. In this narrative review, we integrate current evidence to examine the role of RAD18 in breast cancer radioresistance and to evaluate how RAD18-mediated DNA damage tolerance may be functionally embedded within broader PAM-driven survival programs. While existing data support a biologically plausible convergence between DNA damage tolerance mechanisms and oncogenic survival signaling, direct mechanistic evidence linking RAD18 to PAM pathway activity in breast cancer remains limited. We therefore propose a subtype-informed conceptual framework in which RAD18-dependent stress tolerance operates within a permissive survival signaling context. Such a framework may refine the interpretation of radioresistance and inform the development of predictive biomarkers as well as rational radiosensitization strategies.

## 1 Introduction

Breast cancer represents a substantial global health burden and remains one of the leading causes of cancer-related mortality among women worldwide. In China alone, approximately 357,000 new cases and 75,000 deaths were reported in 2022 [1]. Among patients initially diagnosed with early-stage disease, approximately 20–30 % ultimately develop metastatic progression, whereas 5–10 % present with distant metastases at diagnosis [1]. These data underscore the persistent clinical challenge posed by disease progression and treatment failure.

Current therapeutic strategies for breast cancer encompass surgery, chemotherapy, radiotherapy, endocrine therapy, targeted therapy, and immunotherapy. Among these modalities, radiotherapy represents a cornerstone of treatment, aiming to deliver tumoricidal doses while minimizing damage to surrounding normal tissues [2]. Despite substantial improvements in locoregional control and survival outcomes, intrinsic and acquired resistance to ionizing radiation remains a major clinical obstacle. Radioresistance contributes not only to local recurrence but also to metastatic dissemination, ultimately limiting survival and adversely affecting quality of life [3]. Accordingly, delineating the molecular determinants of radioresistance and identifying actionable radiosensitization strategies remain central priorities in breast cancer research.

Among the signaling networks implicated in therapeutic resistance, the PI3K/AKT/mTOR (PAM) pathway is of particular relevance. This pathway constitutes one of the most frequently dysregulated oncogenic axes in breast cancer and regulates a wide range of cellular processes, including proliferation, survival, metabolism, angiogenesis, and stress adaptation [4]. Its aberrant activation commonly arises from PIK3CA mutations, PTEN loss or inactivation, or dysregulation of upstream receptor tyrosine kinase signaling [4], 5]. Importantly, accumulating evidence indicates that activation of the PAM pathway is consistently associated with resistance to endocrine therapy and targeted agents, and is increasingly implicated in modulating responses to radiotherapy [6]. Mechanistically, PAM signaling integrates pro-survival and stress-adaptive programs, thereby positioning it as a central node in treatment failure [7].

However, the biological and clinical significance of PAM signaling is not uniform across breast cancer subtypes. The most robust clinical and translational evidence has been generated in estrogen receptor–positive (ER+)/hormone receptor-positive (HR+), HER2-negative disease, where pathway-directed therapies have already been successfully incorporated into clinical practice [8]. In contrast, in triple-negative breast cancer (TNBC), the role of PAM signaling appears more heterogeneous and is tightly intertwined with additional processes, including stemness, DNA repair capacity, and tumor microenvironmental regulation [9]. These observations highlight the necessity of interpreting PAM pathway activity within a subtype-specific framework rather than as a universal determinant of resistance.

In parallel with survival signaling networks, increasing attention has been directed toward mechanisms of DNA damage tolerance as contributors to treatment resistance. RAD18, an evolutionarily conserved E3 ubiquitin ligase, plays a central role in translesion DNA synthesis (TLS) and post-replication repair (PRR) [10]. By mediating the monoubiquitination of proliferating cell nuclear antigen (PCNA) in response to replication stress, RAD18 enables replication fork progression across damaged DNA templates, thereby promoting cellular tolerance to genotoxic insults [10]. While this function is essential for maintaining genomic integrity under physiological conditions, it may also permit tumor cells to survive otherwise lethal DNA damage, including that induced by ionizing radiation.

Consistent with this notion, ionizing radiation induces both DNA lesions and replication-associated stress, and RAD18 has been implicated in coordinating damage tolerance with checkpoint-associated survival programs, particularly at the G_2_/M transition, as well as in broader DNA damage response pathways [11]. Elevated RAD18 expression has been reported across multiple malignancies, including breast cancer, colorectal cancer, and glioblastoma, and has been associated with tumor progression, therapeutic resistance, and adverse clinical outcomes [12], [13], [14]. Nevertheless, the specific contribution of RAD18 to breast cancer radioresistance remains incompletely defined, and its integration with oncogenic signaling networks has not been systematically elucidated.

In this review, we integrate current evidence to examine the role of RAD18 in breast cancer radioresistance and to explore its potential interaction with broader survival pathways, with a particular focus on PAM signaling. Given the limited direct mechanistic evidence linking RAD18 to the PI3K/AKT/mTOR axis in breast cancer, we aim to define a conceptual framework that accommodates both DNA damage tolerance and oncogenic survival signaling. By doing so, we seek to clarify how these processes may converge to shape radiotherapy response and to identify key knowledge gaps that warrant further investigation.

To improve conceptual clarity, this review is structured around three key components:

(1) the role of the PAM signaling pathway as a central survival network in breast cancer radioresistance;
(2) the function of RAD18 as a regulator of DNA damage tolerance; 
(3) a proposed framework integrating these processes to explain treatment resistance.

## 2 Methods

This narrative review was conducted through a structured literature search aimed at identifying studies relevant to RAD18, the PI3K/AKT/mTOR (PAM) signaling pathway, and breast cancer radioresistance.

Electronic databases including PubMed, Web of Science, and Scopus were systematically searched. The following keywords and combinations were used: “breast cancer”, “RAD18”, “radioresistance”, “radiotherapy”, “DNA damage tolerance”, “translesion synthesis”, “PI3K/AKT/mTOR”, and “PTEN”.

The search focused primarily on studies published between 2015 and 2025, while earlier seminal studies were included where necessary to provide mechanistic context.

Studies were included if they (i) investigated mechanisms of radioresistance or DNA damage response, (ii) examined RAD18 function or regulation, or (iii) explored PAM pathway signaling in cancer biology or treatment resistance. Studies lacking mechanistic relevance or not peer-reviewed were excluded.

Given the limited number of breast cancer–specific studies on RAD18, evidence from other tumor types was incorporated where appropriate to inform mechanistic interpretation.

## 3 Mechanisms of the PAM signaling pathway in breast cancer radioresistance

### 3.1 The PAM signaling pathway in breast cancer

The PI3K/AKT/mTOR (PAM) signaling pathway represents one of the most frequently dysregulated oncogenic networks in breast cancer and plays a central role in coordinating tumor cell growth, survival, metabolic adaptation, and therapeutic resistance [4]. Aberrant activation of this pathway commonly arises from PIK3CA mutations, PTEN loss or functional inactivation, and dysregulation of upstream receptor tyrosine kinase signaling, all of which converge to sustain constitutive pro-survival signaling [5].

Importantly, the biological and clinical significance of PAM pathway activation is highly context-dependent. The most robust evidence has been established in estrogen receptor-positive (ER+)/hormone receptor-positive (HR+), HER2-negative breast cancer, where pathway alterations are both prevalent and therapeutically actionable [15]. In this setting, pharmacological inhibition of PAM signaling has demonstrated clinical benefit, thereby validating its role as a key driver of treatment resistance. In contrast, in triple-negative breast cancer (TNBC), PAM pathway activity appears more heterogeneous and is frequently integrated with additional regulatory layers, including DNA repair capacity, stemness-associated programs, and microenvironmental signaling [9].

These observations underscore an important conceptual point: PAM signaling should not be interpreted as a uniform determinant of resistance across all breast cancers. Rather, its contribution must be evaluated within a subtype-specific biological context. This consideration is particularly relevant when examining its potential role in modulating radiotherapy response.

### 3.2 PAM signaling pathway as a contributor to radioresistance

Radiotherapy exerts its antitumor effects primarily through the induction of DNA damage, replication stress, and associated cellular injury [2]. Within this context, aberrant activation of the PAM signaling pathway may promote a radioresistant phenotype through multiple, mechanistically interconnected processes [16].

First, PAM signaling enhances tumor cell survival by suppressing apoptosis and promoting pro-survival signaling cascades, thereby enabling cells to withstand radiation-induced cytotoxic stress [17]. Second, accumulating evidence suggests that this pathway may facilitate DNA damage recovery and checkpoint adaptation following irradiation, allowing tumor cells to resume proliferation despite persistent genomic lesions [18]. Third, downstream signaling through mTOR has been implicated in the regulation of autophagy, metabolic reprogramming, and the maintenance of cancer stem–like properties [19], all of which may support cellular persistence under conditions of radiation-induced stress.

Rather than operating through a single linear cascade, the PAM pathway functions as an integrated adaptive network. At the upstream level, PI3K activation may be driven by receptor tyrosine kinase signaling or by genomic alterations such as PIK3CA mutation and PTEN loss [20], 21]. AKT, as a central signaling hub, integrates survival, metabolic, and anti-apoptotic signals, thereby coordinating cellular responses to stress [4]. mTOR, in turn, amplifies resistance-associated phenotypes by regulating protein synthesis, cell growth, and metabolic adaptation [22]. Collectively, these interconnected nodes enable irradiated tumor cells to evade cell death and maintain viability under genotoxic conditions.

Importantly, this interpretation is consistent with broader observations in breast cancer therapy resistance. In HR+/HER2− disease, dysregulation of PAM signaling has been firmly established as a driver of resistance to endocrine therapy and CDK4/6 inhibitors, providing a clinically validated framework for its role in adaptive survival [8]. In TNBC, although the clinical evidence remains less mature, PAM pathway activation has been associated with aggressive tumor behavior and poor prognosis, albeit within a more biologically heterogeneous context [9].

Taken together, these findings support the notion that PAM signaling does not merely contribute to resistance through isolated mechanisms but instead acts as a central integrative network that coordinates survival, stress adaptation, and recovery following therapeutic insult, including irradiation.

### 3.3 Implications for a RAD18-centered interpretation

The inclusion of the PAM signaling pathway in this review should not be interpreted as suggesting that it independently accounts for all aspects of breast cancer radioresistance. Rather, its relevance lies in its capacity to define the cellular context within which DNA damage tolerance mechanisms operate.

In this framework, RAD18-dependent processes – such as translesion synthesis, replication stress tolerance, and post-irradiation survival – may be functionally embedded within a broader PAM-driven pro-survival environment. In other words, while RAD18 may directly mediate the processing and tolerance of DNA damage, PAM signaling may concurrently establish a permissive cellular state that enables these processes to translate into sustained tumor cell survival.

At present, direct mechanistic links between RAD18 and the PAM axis in breast cancer remain insufficiently characterized. Existing evidence supports a biologically plausible intersection between DNA damage tolerance, checkpoint adaptation, and survival signaling; however, whether RAD18 functionally interacts with, or is regulated by, PAM pathway components in the context of radiotherapy response has not been conclusively demonstrated.

Accordingly, a more precise conceptual interpretation is that RAD18 and PAM signaling may operate in parallel, or potentially converge at the level of stress adaptation and survival, rather than forming a currently established linear signaling axis. This distinction is critical to avoid overinterpretation of limited data while still acknowledging a biologically coherent hypothesis.

From a translational perspective, this conceptual framework has important implications. It suggests that effective radiosensitization strategies may require simultaneous targeting of DNA damage tolerance mechanisms and oncogenic survival signaling, particularly in molecular contexts characterized by high PAM pathway activity. However, such strategies will require rigorous experimental validation, ideally within subtype-specific models that reflect the biological heterogeneity of breast cancer.

## 4 RAD18 in breast cancer

### 4.1 Biological rationale for studying RAD18

RAD18 is an evolutionarily conserved E3 ubiquitin ligase that occupies a central position in DNA damage tolerance and the maintenance of genomic stability [23]. Functionally, RAD18 operates in conjunction with the E2 ubiquitin-conjugating enzyme RAD6 (HHR6A/HHR6B in humans) to mediate the monoubiquitination of proliferating cell nuclear antigen (PCNA), a key molecular switch that facilitates translesion DNA synthesis (TLS) and post-replication repair (PRR) [24]. Through this mechanism, RAD18 enables replication fork progression across damaged DNA templates, thereby preventing fork collapse and sustaining DNA synthesis under conditions of replication stress.

Importantly, RAD18-mediated processes are conceptually distinct from canonical high-fidelity DNA repair pathways. Rather than directly correcting DNA lesions, RAD18 promotes damage tolerance, allowing cells to bypass unrepaired DNA damage at the cost of increased mutagenesis. This functional distinction is critical in the context of cancer, where tolerance of DNA damage may provide a selective survival advantage under genotoxic stress. Thus, RAD18 can be viewed not merely as a component of the DNA damage response, but as a regulator of the balance between genome maintenance and cellular survival.

In the setting of radiotherapy, this distinction becomes particularly relevant. Ionizing radiation induces a spectrum of DNA lesions and replication-associated stress that threaten cell viability. The ability of tumor cells to survive such stress depends not only on efficient DNA repair but also on their capacity to tolerate and bypass DNA damage. In this context, RAD18 may function as a molecular integrator that links replication stress responses, damage tolerance, and post-damage survival.

However, the role of RAD18 is inherently context-dependent. While physiological RAD18 activity contributes to genome stability in normal cells, its sustained or dysregulated activation in cancer cells may facilitate continued proliferation under genotoxic conditions, thereby promoting therapeutic resistance. This dual role highlights the importance of distinguishing between homeostatic and oncogenic functions of RAD18 when interpreting its contribution to radiotherapy response.

### 4.2 RAD18 expression and its potential relevance to breast cancer progression

Accumulating evidence indicates that RAD18 is aberrantly expressed across multiple human malignancies and may be associated with aggressive tumor phenotypes [25], 26]. In breast cancer, analyses of publicly available datasets suggest that RAD18 expression is elevated in tumor tissues relative to normal controls and may increase with disease progression, although these observations require further validation in well-controlled clinical cohorts.

Clinically, elevated RAD18 expression has been associated with unfavorable outcomes in several cancer types, raising the possibility that RAD18 may have prognostic relevance. However, its significance in breast cancer appears to be context-dependent rather than uniform across subtypes. For example, in advanced triple-negative breast cancer (TNBC), RAD18 has been implicated in a proposed RAD18–YAP–TGF-β positive feedback loop involving M2-polarized tumor-associated macrophages, which may enhance tumor stemness and promote disease progression [13]. Although this model remains to be independently validated, it is noteworthy in that it positions RAD18 within a broader regulatory network linking DNA damage tolerance, microenvironmental signaling, and tumor plasticity.

More broadly, these observations support the notion that RAD18 may contribute not only to DNA damage tolerance but also to tumor aggressiveness in specific biological contexts. Nevertheless, current evidence remains largely correlative, and the extent to which RAD18 directly drives tumor progression – as opposed to serving as a marker of underlying genomic stress or proliferative activity – remains unclear. Consequently, RAD18 should be considered a candidate biomarker with emerging but not yet definitive clinical relevance in breast cancer.

### 4.3 RAD18 as a potential mediator of radioresistance

The G2/M checkpoint represents a critical cell-cycle control mechanism that prevents cells harboring damaged or incompletely replicated DNA from entering mitosis [27]. In irradiated tumor cells, activation of this checkpoint provides a temporal window that facilitates DNA damage processing and promotes survival. Checkpoint adaptation, in this context, is widely recognized as a key determinant of radioresistance.

RAD18 is of particular interest within this framework because it resides at the interface between DNA damage tolerance, replication stress responses, and checkpoint-associated survival. Following irradiation, RAD18 is recruited to stalled replication forks, where it promotes PCNA monoubiquitination and facilitates lesion bypass through translesion synthesis. By enabling replication to proceed in the presence of DNA damage, RAD18 may reduce replication-associated catastrophe and support continued cell viability under genotoxic stress.

In addition to its role in replication-associated processes, RAD18 has been implicated in the coordination of checkpoint signaling. Experimental evidence suggests that RAD18 may contribute to G2/M checkpoint regulation and thereby enhance the ability of tumor cells to withstand DNA damage prior to mitotic entry [11]. Consistent with this notion, RAD18 deficiency has been reported to increase radiosensitivity in certain model systems, supporting a functional role in promoting survival following irradiation.

Notably, radioresistant breast cancer cells frequently exhibit enhanced G2/M-phase arrest in conjunction with activation of pro-survival signaling pathways, including the PAM axis, as reflected by increased levels of phosphorylated AKT and mTOR [28]. This observation raises the possibility that RAD18-mediated DNA damage tolerance may operate in concert with oncogenic survival signaling to reinforce resistance phenotypes.

**Figure 1:**
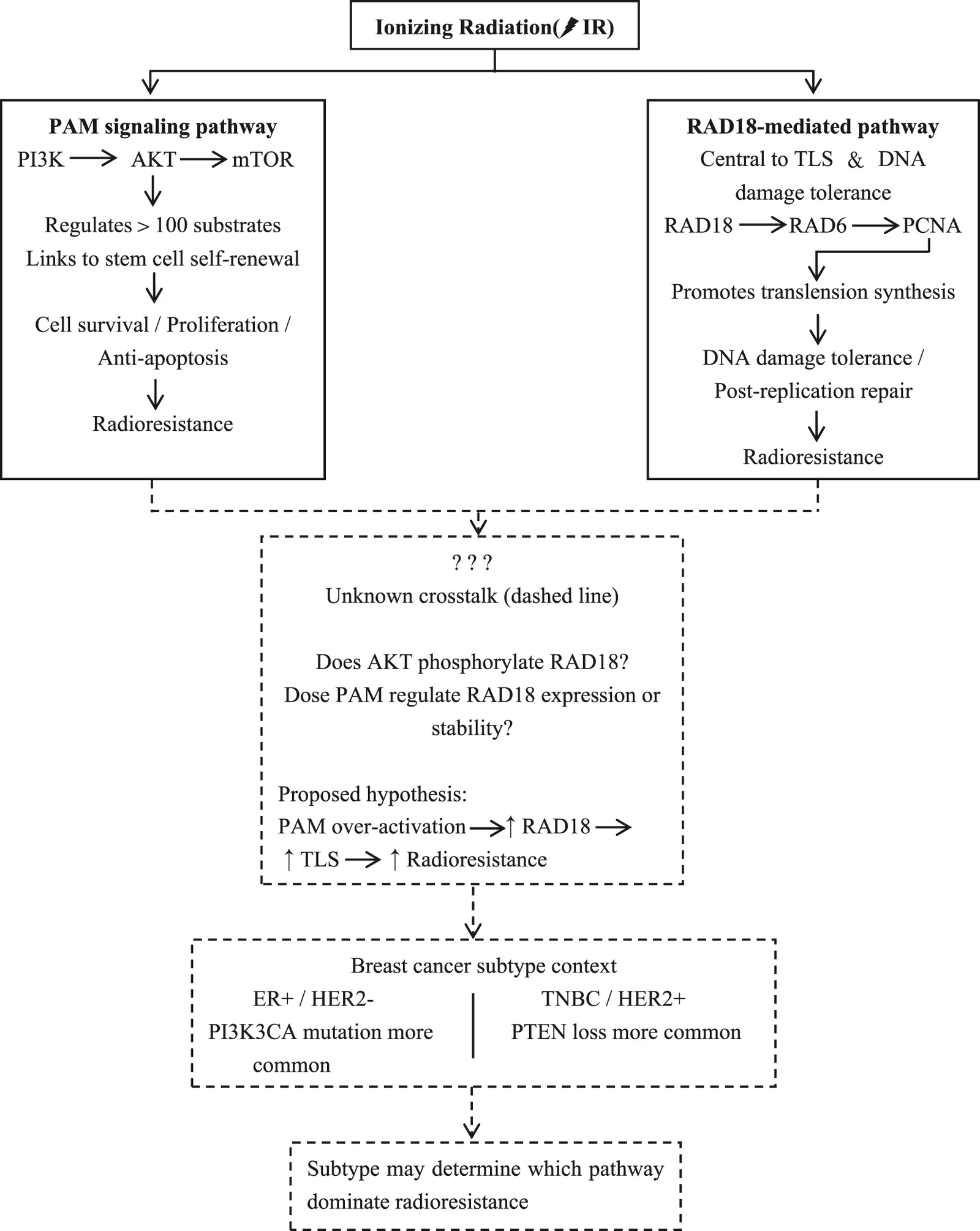
Proposed crosstalk between the PAM signaling pathway and RAD18 in breast cancer radioresistance: current evidence and knowledge gaps.

However, it is important to emphasize that direct mechanistic evidence linking RAD18 activity to radioresistance in breast cancer remains limited. While available data support a plausible role for RAD18 in facilitating post-irradiation survival, the precise molecular pathways through which it exerts this effect – and whether these pathways are subtype-specific – have not been fully elucidated. Accordingly, RAD18 should currently be regarded as a biologically plausible, yet not fully validated, mediator of breast cancer radioresistance (Figure 1).

## 5 Discussion

### 5.1 RAD18 as a candidate mediator of tumor radioresistance

Radiotherapy exerts its antitumor effects primarily through the induction of extensive DNA damage, including both double-strand breaks and replication-associated lesions. In response to such genotoxic stress, RAD18 is recruited to stalled replication forks, where it forms a functional complex with the ubiquitin-conjugating enzyme RAD6 and promotes proliferating cell nuclear antigen (PCNA) monoubiquitination, thereby facilitating post-replication repair and translesion synthesis [29]. Through these activities, RAD18 enables tumor cells to tolerate otherwise cytotoxic DNA lesions, potentially reducing apoptosis and enhancing survival following irradiation.

Evidence from multiple tumor types supports a role for RAD18 in treatment resistance. In esophageal squamous cell carcinoma, elevated RAD18 expression has been associated with poor prognosis in patients receiving radiotherapy, and mechanistic studies suggest that RAD18 may reduce radiosensitivity through upregulation of p-DNA-PKcs [30]. In glioma, RAD18 has been reported to modulate p53-related signaling pathways, thereby promoting proliferation and suppressing apoptosis in response to radiation [31]. In addition, RAD18 downregulation has been shown to activate caspase-9/caspase-3–dependent apoptosis and enhance treatment sensitivity in rectal cancer models [25].

Collectively, these findings support a broader interpretation of RAD18 as a stress-response factor that enables tumor cell persistence under genotoxic therapy. However, breast cancer–specific evidence remains limited. Although elevated RAD18 expression has been associated with poor prognosis and increased radioresistance in breast cancer [13], the precise molecular mechanisms through which RAD18 contributes to this phenotype remain incompletely defined. Accordingly, RAD18 should currently be regarded as a biologically plausible, yet not fully validated, mediator of radioresistance in breast cancer.

### 5.2 Potential links between RAD18 and PAM signaling

Following exposure to ionizing radiation, RAD18 participates in DNA damage tolerance and contributes to the coordination of checkpoint control and genomic stability [13]. Beyond its canonical role, emerging evidence from non-breast cancer models suggests that RAD18 may also intersect with signaling pathways that regulate proliferation and survival. For instance, in melanoma, RAD18 expression peaks during the G2/M phase and has been linked to cyclin D1 regulation and proliferative signaling [32]. In glioma, RAD18 knockdown has been associated with reduced viability, increased apoptosis, and suppression of migration and invasion, accompanied by enrichment of genes involved in survival-related signaling pathways [33].

While these observations raise the possibility that RAD18 may interface with oncogenic signaling networks, they do not directly establish a mechanistic link between RAD18 and the PI3K/AKT/mTOR pathway in breast cancer. A more cautious interpretation is that RAD18-mediated DNA damage tolerance may operate in parallel with, or converge upon, survival-promoting signaling axes such as PAM.

Importantly, the PAM pathway itself is a central regulator of proliferation, metabolism, and stress adaptation in breast cancer, and its dysregulation has been widely documented across molecular subtypes [34], 35]. From a systems-level perspective, it is therefore plausible that RAD18-dependent tolerance mechanisms may be functionally embedded within a broader pro-survival signaling context defined by PAM activation. However, whether RAD18 directly interacts with PAM components, is transcriptionally or post-translationally regulated by this pathway, or contributes to its downstream effects remains to be determined.

Taken together, current evidence supports a model of functional convergence rather than a defined linear signaling axis, highlighting an important area for future mechanistic investigation.

### 5.3 PTEN loss as a permissive context for RAD18-associated radioresistance

PTEN is a critical tumor suppressor that negatively regulates the PAM signaling pathway [21]. Loss of PTEN function is frequently observed in breast cancer and is associated with tumor progression, metastasis, and resistance to therapy [36]. In PTEN-deficient contexts, hyperactivation of the PAM pathway enhances pro-survival signaling and promotes adaptive resistance phenotypes [5].

More broadly, dysregulation of the PAM axis – including PTEN loss, PI3K activation, and AKT gain-of-function – represents a common molecular feature across breast cancer subtypes and defines an important therapeutic landscape [34]. Previous studies have suggested that PTEN-deficient cells may acquire survival advantages through downstream signaling cascades, and that pharmacological inhibition of PAM components can partially restore treatment sensitivity in these settings [37], 38].

Notably, prior mechanistic work has indicated that PCNA ubiquitination may be regulated through an AKT/REV1/RAD18-related axis following irradiation [39], and that REV1 may further enhance this process through interaction with RAD18 [40]. These observations are particularly intriguing, as they suggest a potential molecular interface between DNA damage tolerance and oncogenic survival signaling.

However, it is important to emphasize that direct evidence demonstrating that RAD18 promotes breast cancer radioresistance through a PTEN-dependent PAM mechanism is currently lacking. Accordingly, we propose this relationship as a working hypothesis: RAD18-mediated DNA damage tolerance may be particularly advantageous in tumor cells that exist within a PAM-activated, pro-survival context, such as PTEN-deficient breast cancers. This hypothesis provides a testable framework for future studies but should not be interpreted as a confirmed mechanistic pathway.

### 5.4 A conceptual model and implications for future research

Based on the available evidence, we propose a conceptual model in which breast cancer radioresistance arises from the functional convergence of DNA damage tolerance and oncogenic survival signaling. In this model, RAD18 acts as a key mediator of replication stress tolerance and lesion bypass, enabling tumor cells to survive DNA damage induced by radiotherapy. Concurrently, activation of the PAM pathway establishes a pro-survival cellular state characterized by enhanced anti-apoptotic signaling, metabolic adaptation, and stress resilience.

Within this framework, neither RAD18 nor PAM signaling alone is likely sufficient to fully account for radioresistance. Rather, their combined activity may create a permissive environment in which tumor cells can both tolerate DNA damage and sustain viability. This model provides a mechanistic rationale for the observed association between DNA damage response pathways and survival signaling in resistant tumors.

Importantly, this conceptual framework generates several testable hypotheses. First, the contribution of RAD18 to radioresistance may be subtype-specific, depending on the degree of PAM pathway activation. Second, RAD18 expression and function may be modulated by oncogenic signaling, either directly or indirectly. Third, combined targeting of RAD18-mediated DNA damage tolerance and PAM signaling may produce synergistic radiosensitizing effects, particularly in molecular contexts characterized by pathway hyperactivation.

Future research should therefore focus on (i) defining the subtype-specific role of RAD18 in breast cancer, (ii) elucidating potential mechanistic interactions between RAD18 and PAM pathway components, and (iii) evaluating combination strategies that simultaneously disrupt DNA damage tolerance and survival signaling. Such studies will be essential to determine whether RAD18 can be translated from a candidate mediator into a clinically actionable biomarker or therapeutic target.

### 5.5 Limitations

Several limitations should be acknowledged. First, direct mechanistic evidence linking RAD18 to radioresistance in breast cancer remains limited, and some conclusions are extrapolated from studies in other tumor types. Second, the heterogeneity of breast cancer subtypes complicates interpretation of both RAD18 function and PAM pathway activity. Third, as a narrative review, this study may be influenced by selection bias in the available literature.

Future studies should therefore prioritize subtype-specific validation and experimental confirmation of the proposed mechanisms.

### 5.6 Proposed hypothesis

RAD18-mediated DNA damage tolerance confers a selective survival advantage in breast cancer cells with hyperactivated PAM signaling, such as those harboring PTEN loss or PI3K pathway alterations. This interaction represents a context-dependent mechanism of radioresistance and may provide a rationale for combined therapeutic targeting.

## 6 Conclusions

In summary, RAD18 represents a biologically plausible contributor to breast cancer radioresistance due to its central role in DNA damage tolerance, replication stress adaptation, and checkpoint-associated survival. Concurrently, the PI3K/AKT/mTOR pathway constitutes a key, subtype-dependent survival network that shapes therapeutic response in breast cancer.

Although a direct mechanistic interaction between RAD18 and PAM signaling in breast cancer has not yet been conclusively established, their potential functional convergence provides a coherent framework for understanding radioresistance. Importantly, both RAD18 activity and PAM signaling must be interpreted within the context of molecular subtype rather than as uniform determinants across all breast cancers.

Future studies should prioritize subtype-specific validation and mechanistic dissection of the RAD18–PAM interface, with particular emphasis on ER-positive and triple-negative disease. A deeper understanding of how DNA damage tolerance intersects with oncogenic survival signaling may ultimately enable the identification of novel biomarkers and the development of rational radiosensitization strategies to improve clinical outcomes in breast cancer.

## References

[1] The Breast Cancer Professional Committee of the Chinese Anti-Cancer Association and the Breast Oncology Group of the Oncology Branch of the Chinese Medical Association (2025). “Guidelines and specifications for the diagnosis and treatment of breast cancer” of the Chinese Anti-Cancer Association (2026 Edition). Chin J Cancer.

[2] Costin IC, Marcu LG (2022). Factors impacting on patient setup analysis and error management during breast cancer radiotherapy. Crit Rev Oncol Hematol.

[3] Duma MN, ReneBudach WD, JuergenFeyer PF, RainerHaase W, WolfgangHehr TK, DavidPiroth MS (2019). Heart-sparing radiotherapy techniques in breast cancer patients: a recommendation of the breast cancer expert panel of the German society of radiation oncology (DEGRO). Strahlenther Onkol.

[4] Wanigasooriya K, Tyler R, Barros-Silva JD, Sinha Y, Ismail T, Beggs AD (2020). Radiosensitising cancer using phosphatidylinositol-3-kinase (PI3K), protein kinase B (AKT) or mammalian target of rapamycin (mTOR) inhibitors. Cancers (Basel).

[5] Miricescu D, Totan A, Stanescu-Spinu II, Badoiu SC, Greabu M (2020). PI3K/AKT/mTOR signaling pathway in breast cancer: from molecular landscape to clinical aspects. Int J Mol Sci.

[6] Ma F, Xu BH (2022). PI3K/AKT/Mtor expert consensus on the clinical application of signal pathway inhibitors in the treatment of breast cancer. Chin J Oncol.

[7] Browne IM, Okines AFC (2024). Resistance to targeted inhibitors of the PI3K/AKT/mTOR pathway in advanced Oestrogen-receptor-positive breast cancer. Cancers (Basel).

[8] Hao C, Wei Y, Meng W, Zhang J, Yang X (2025). PI3K/AKT/mTOR inhibitors for hormone receptor-positive advanced breast cancer. Cancer Treat Rev.

[9] Zhang HP, Jiang RY, Zhu JY, Sun KN, Huang Y, Zhou HH (2024). PI3K/AKT/mTOR signaling pathway: an important driver and therapeutic target in triple-negative breast cancer. Breast Cancer.

[10] Masuda Y, Suzuki M, Kawai H, Kamiya K (2012). Asymmetric nature of two subunits of RAD18, a RING-type ubiquitin ligase E3, in the human RAD6A–RAD18 ternary complex. Nucleic Acids Res.

[11] Sasatani M, Xu Y, Hidehiko K, Cao L, Satoshi T, Tsutomu S (2015). RAD18 activates the G2/M checkpoint through DNA damage signaling to maintain genome integrity after ionizing radiation exposure. PLoS One.

[12] Xie C, Wang HW, Cheng HB, Li JH, Wang Z, Yue W (2014). RAD18 mediates resistance to ionizing radiation in human glioma cells. Biochem Biophys Res Commun.

[13] Yan XQ, He YZ, Yang SK, Zeng TY, Hua YZ, Bao SN (2022). A positive feedback loop: RAD18-YAP-TGF-β between triple-negative breast cancer and macrophages regulates cancer stemness and progression. Cell Death Discov.

[14] Li P, He C, Gao A, Yan X, Xia X, Zhou J (2020). RAD18 promotes colorectal cancer metastasis by activating the epithelial-mesenchymal transition pathway. Oncol Rep.

[15] Ferro A, Campora M, Caldara A, De Lisi D, Lorenzi M, Monteverdi S (2024). Novel treatment strategies for hormone receptor (HR)-positive, HER2-negative metastatic breast cancer. J Clin Med.

[16] Chen YH, Wei MF, Wang CW, Lee HW, Pan SL, Gao M (2015). Dual phosphoinositide 3-kinase/mammalian target of rapamycin inhibitor is an effective radiosensitizer for colorectal cancer. Cancer Lett.

[17] Choi YJ, Anders L (2014). Signaling through cyclin D-dependent kinases. Oncogene.

[18] Luo X, Wei YQ, Lin HX, Xiao N, Zhao W (2025). BUB1B promotes homologous recombination-mediated DNA damage repair in breast cancer cells through the PI3K/AKT signaling pathway. Oncol Rep.

[19] Browne IM, André F, Chandarlapaty S, Carey LA, Turner NC (2024). Optimal targeting of PI3K-AKT and mTOR in advanced oestrogen receptor-positive breast cancer. Lancet Oncol.

[20] López-Knowles E, O’Toole S, Mcneil C, Millar E, Qiu M, Crea P (2010). PI3K pathway activation in breast cancer is associated with the basal-like phenotype and cancer-specific mortality. Int J Cancer.

[21] Hopkins BD, Hodakoski C, Douglas B, Mense M (2014). PTEN function: the long and the short of it. Trends Biochem Sci.

[22] Nam HY, Han MW, Chang HW, Kim SY, Kim SW (2013). Prolonged autophagy by MTOR inhibitor leads radioresistant cancer cells into senescence. Autophagy.

[23] Xin H (2000). The human RAD18 gene product interacts with HHR6A and HHR6B. Nucleic Acids Res.

[24] Tateishi S, Niwa H, Miyazaki JI, Fujimoto S, Inoue H, Yamaizumi M (2003). Enhanced genomic instability and defective postreplication repair in RAD18 knockout mouse embryonic stem cells. Mol Cell Biol.

[25] Yan X, Chen J, Meng Y, He C, Zhou J, Li P (2019). RAD18 may function as a predictor of response to preoperative concurrent chemoradiotherapy in patients with locally advanced rectal cancer through caspase‐9‐caspase‐3‐dependent apoptotic pathway. Cancer Med.

[26] Lou J, Yang Y, Gu Q, Price BA, Wu D, Fedoriw Y (2021). Rad18 mediates specific mutational signatures and shapes the genomic landscape of carcinogen-induced tumors in vivo. NAR Cancer.

[27] Verkade HM, Bugg SJ, Lindsay HD, Carr AM, O’Connell MJ (1999). Rad18 is required for DNA repair and checkpoint responses in fission yeast. Mol Biol Cell.

[28] Lee H, Park J, Jung K, Lim JH, Hong SS (2022). HS-173, a novel PI3K inhibitor enhances radiosensitivity of breast cancer cells. Int J Radiat Res.

[29] Tsuji Y, Watanabe K, Araki K, Shinohara M, Yamagata Y, Tsurimoto T (2008). Recognition of forked and single‐stranded DNA structures by human RAD18 complexed with RAD6B protein triggers its recruitment to stalled replication forks. Genes Cells.

[30] Li X, Zou S, Zhou L, Gao A, Xu J, He C (2022). RAD18 confers radioresistance of esophagus squamous cell carcinoma through regulating p-DNA-PKcs. Cancer Med.

[31] Baatar S, Bai T, Yokobori T, Gombodorj N, Saeki H, Ubukata Y (2020). High RAD18 expression is associated with disease progression and poor prognosis in patients with gastric cancer. Ann Surg Oncol.

[32] Wong RP, Aguissa-Touré AH, Wani AA, Khosravi S, Martinka M, Martinka M (2012). Elevated expression of Rad18 regulates melanoma cell proliferation. Pigment Cell Melanoma Res.

[33] Xie C, Lu D, Xu M, Qu Z, Zhang W, Wang H (2019). Knockdown of RAD18 inhibits glioblastoma development. J Cell Physiol.

[34] Wali AF, Talath S, El Tanani M, Rashid Rangraze I, Babiker R, Shafi S (2025). PI3K/AKT/mTOR pathway in breast cancer pathogenesis and therapy: insights into phytochemical-based therapeutics. Nutr Cancer.

[35] Xiao W, Zhang G, Chen B, Liao N, Wen L, Lai J (2021). Mutational landscape of PI3K-AKT-mTOR pathway in breast cancer: implications for targeted therapeutics. J Cancer.

[36] Fath MK, Ebrahimi M, Nourbakhsh E, Hazara AZ, Mirzaei A, Shafieyari S (2022). PI3K/Akt/mTOR signaling pathway in cancer stem cells. Pathol Res Pract.

[37] Zhang G, Wang WJ, Yao CX, Zhang SP, Liang LL, Han MY (2017). Radiation-resistant cancer stem-like cell properties are regulated by PTEN through the activity of nuclear β-catenin in nasopharyngeal carcinoma. Oncotarget.

[38] Bergholz JS, Wang QW, Wang Q, Ramseier M, Prakadan S, Wang WH (2023). PI3Kβ controls immune evasion in PTEN-deficient breast tumours. Nature.

[39] Villafañez F, García IA, Carbajosa S, Pansa MF, Sabrina M, Llorens MC (2019). AKT inhibition impairs PCNA ubiquitylation and triggers synthetic lethality in homologous recombination-deficient cells submitted to replication stress. Oncogene.

[40] Wang Z, Huang M, Ma X, Li H, Tang T, Guo C (2016). REV1 promotes PCNA monoubiquitylation through interacting with ubiquitylated RAD18. J Cell Sci.

